# Supplementary material for: Empirical methods for controlling false positives and estimating confidence in ChIP-Seq peaks
Source: BMC Bioinformatics. 2008 Dec 5;9:523. doi: 10.1186/1471-2105-9-523 (PMC2628906; doi:10.1186/1471-2105-9-523)
Supplement: Additional File 1 — A variety of html documents from the USeq web site detailing the available applications, their best usage, output file type descriptions, command line menus, etc. [file 1471-2105-9-523-S1.zip › USeqUserGuides/JavaDoc.zip!/JavaDoc/trans/graphics/Box.html]

Box


|  |  |  |
| --- | --- | --- |
| **Overview** \| **Package** \| **Class** \| **Use** \| **Tree** \| **Deprecated** \| **Index** \| **Help** | |  |
| **PREV CLASS**   **NEXT CLASS** | **FRAMES**    **NO FRAMES**     **All Classes** |  |
| SUMMARY: NESTED \| FIELD \| CONSTR \| METHOD | DETAIL: FIELD \| CONSTR \| METHOD |  |


---


## trans.graphics Class Box

```
java.lang.Object
  trans.graphics.Box
```

---

``` public class Box extends java.lang.Object ```

Box for `IntervalPlotter`.

---

**Constructor Summary**

`Box(double baseStart,
double baseStop,
double baseFirstPosition,
java.awt.Color color)`


| **Method Summary** | |
| --- | --- |
| `void` | `draw(java.awt.Graphics2D g2)` |
| `double` | `makeGlyphs(Graph graph, double y)` |

**Methods inherited from class java.lang.Object**

`equals, getClass, hashCode, notify, notifyAll, toString, wait, wait, wait`

**Constructor Detail**

### Box

```
public Box(double baseStart,
           double baseStop,
           double baseFirstPosition,
           java.awt.Color color)
```


**Method Detail**

### makeGlyphs

```
public double makeGlyphs(Graph graph,
                         double y)
```

---


### draw

```
public void draw(java.awt.Graphics2D g2)
```


---


|  |  |  |
| --- | --- | --- |
| **Overview** \| **Package** \| **Class** \| **Use** \| **Tree** \| **Deprecated** \| **Index** \| **Help** | |  |
| **PREV CLASS**   **NEXT CLASS** | **FRAMES**    **NO FRAMES**     **All Classes** |  |
| SUMMARY: NESTED \| FIELD \| CONSTR \| METHOD | DETAIL: FIELD \| CONSTR \| METHOD |  |


---
